# Bioenergetic Alterations of Metabolic Redox Coenzymes as NADH, FAD and FMN by Means of Fluorescence Lifetime Imaging Techniques

**DOI:** 10.3390/ijms22115952

**Published:** 2021-05-31

**Authors:** Sviatlana Kalinina, Christian Freymueller, Nilanjon Naskar, Bjoern von Einem, Kirsten Reess, Ronald Sroka, Angelika Rueck

**Affiliations:** 1 Core Facility Confocal and Multiphoton Microscopy, Ulm University, Albert-Einstein-Allee 11, 89081 Ulm, Germany; nilanjon.naskar@uni-ulm.de (N.N.); kirsten.reess@uni-ulm.de (K.R.); 2 Laser-Forschungslabor, LIFE Center, University Hospital, LMU Munich, Fraunhoferstrasse 20, 82152 Planegg, Germany; christian.freymueller@med.uni-muenchen.de (C.F.); ronald.sroka@med.uni-muenchen.de (R.S.); 3 Department of Urology, University Hospital, LMU Munich, Marchioninistraße 15, 81377 Munich, Germany; 4 Zentrum Biomedizinische Forschung (ZBMF), Department of Neurology, Ulm University, Helmholtzstrasse, 8/1, 89081 Ulm, Germany; bjoern.von-einem@uni-ulm.de

**Keywords:** FLIM, NAD(P)H, FAD, FMN, NAD(P)H metabolic index, FLIRR index, extended FLIRR, cell metabolism, OXPHOS, glycolysis

## Abstract

Metabolic FLIM (fluorescence lifetime imaging) is used to image bioenergetic status in cells and tissue. Whereas an attribution of the fluorescence lifetime of coenzymes as an indicator for cell metabolism is mainly accepted, it is debated whether this is valid for the redox state of cells. In this regard, an innovative algorithm using the lifetime characteristics of nicotinamide adenine dinucleotide (phosphate) (NAD(P)H) and flavin adenine dinucleotide (FAD) to calculate the fluorescence lifetime induced redox ratio (FLIRR) has been reported so far. We extended the FLIRR approach and present new results, which includes FLIM data of the various enzymes, such as NAD(P)H, FAD, as well as flavin mononucleotide (FMN). Our algorithm uses a two-exponential fitting procedure for the NAD(P)H autofluorescence and a three-exponential fit of the flavin signal. By extending the FLIRR approach, we introduced FLIRR1 as protein-bound NAD(P)H related to protein-bound FAD, FLIRR2 as protein-bound NAD(P)H related to free (unbound) FAD and FLIRR3 as protein-bound NAD(P)H related to protein-bound FMN. We compared the significance of extended FLIRR to the metabolic index, defined as the ratio of protein-bound NAD(P)H to free NAD(P)H. The statistically significant difference for tumor and normal cells was found to be highest for FLIRR1.

## 1. Introduction

A common property during tumor development and other diseases is altered energy metabolism, which can lead to a switch between oxidative phosphorylation (OXPHOS) and a glycolytic profile. Fluorescence lifetime imaging (FLIM) of metabolic coenzymes i.e., NAD(P)H (nicotinamide adenine dinucleotide (phosphate)) and FAD (flavin adenine dinucleotide), is now widely accepted to be one of the most important methods for metabolic imaging. It could be demonstrated that time-correlated single photon counting (TCSPC) techniques can separate different decaying compounds with high spatial and temporal resolution [1]. Various algorithms are developed to get reproducible and convincing results (for review see [2]). The correct interpretation of the cellular redox state and the correlation with the fluorescence lifetime τ of the coenzymes is, however, still debated. In 1979, Britton Chance replaced the biochemically defined redox ratio of NAD(P)H related to NAD^+^ to a measure, defined as NAD(P)H related to FAD^+^, the so-called optical redox ratio which uses only fluorescent parameters [3]. This intensity-based optical redox ratio determines the redox ratio of cells and distinguishes oxidized from reduced states. A correlation with the fluorescence lifetime is valid only for special conditions if the NADH/NAD^+^ pool is stable [3]. However, NAD^+^ can be metabolized in different reactions, leading to a change in the redox ratio but not necessarily in the fluorescence lifetime, which is only an indicator of cell metabolism [4,5,6]. In contrast, a change in the NADH/NAD^+^ ratio which affects the binding dynamics of NADH-related enzymes can change the lifetime components of NADH [7,8]. Therefore, a careful interpretation of the redox state is needed when comparing the fluorescence lifetime of different cell systems. New algorithms are needed to circumvent these problems, and to image cell metabolism and redox state from fluorescence lifetimes in complex cellular systems. In this regard, results published so far will be summarized and new approaches will be discussed. In this work, the significance of a metabolic index based on NAD(P)H FLIM will be explained and the results will be compared to the fluorescence lifetime induced redox ratio (FLIRR) where FLIM parameters of NAD(P)H and FAD are related [9]. In contrast to invasive biochemical methods, including chromatography analysis, the optical techniques of measuring the metabolic index based on NAD(P)H FLIM, as well as FLIRR is a non-invasive straightforward and direct technique, allowing on-line visualization of the redox state.

**Figure 1:**
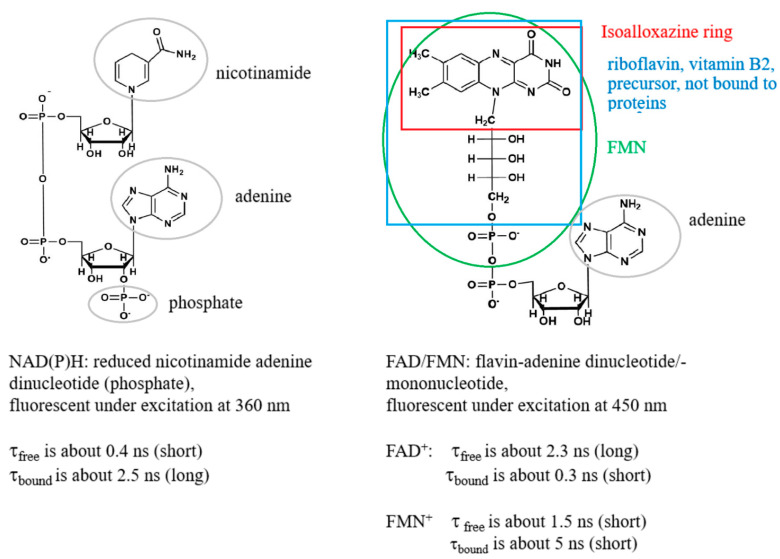
Intracellular coenzymes that play key roles in energy metabolism and metabolic FLIM and their spectral and lifetime characteristics obtained from published data [10,12,13,14,15]. The figure was prepared using the software “ChemDraw (version 20.1.0.110)”.

Coenzymes that are involved in energy metabolism belong to the NAD(P)H and flavin family, as FAD and FMN (flavin mononucleotide). Their spectral and lifetime characteristics that are found in the literature [10,11,12,13,14,15] are the results of their chemical structures summarized in Figure 1.

Binding of NADH to proteins of the respiratory chain during OXPHOS increases the proportion of the protein-bound enzyme, which possesses a longer fluorescence lifetime, whereas a decreased portion of the protein-bound NADH and increased free NADH during anaerobic glycolysis induces a shortening of the fluorescence lifetime (see Figure 1 and references [1,8,12]). Due to both free and bound NADH being found simultaneously, a biexponential fit of the fluorescence decay is normally performed. By inspecting the ratio of the exponential coefficients (amplitudes) of the short and long lifetime components (a_1_/a_2_) a change from OXPHOS to glycolytic cellular states can be observed during tumor development [16,17]. A 10% increase of the ratio was significant to observe the metabolic switch [17]. Similarly, cisplatin induced a decrease of the ratio by approx. 10% in HeLa tumor, indicating a switch from glycolysis to OXPHOS, correlated with apoptosis [18]. The ratio a_1_/a_2_, the so-called metabolic index based on NAD(P)H FLIM, seems to be a useful metric to diagnose cell metabolism. However, this parameter is also not fully correlated to the redox state of the cells. Following the idea of Britton Chance and his definition of the optical redox ratio, FAD should be included in the calculations. As a consequence, quantitative optical metabolic imaging was introduced by Walsh et al., which depends on both the intensity based redox ratio and the fluorescence lifetimes of NAD(P)H and FAD [19]. Moreover, Periasamy and coworkers defined the FLIRR index where the relative amount of bound NAD(P)H is correlated with the relative amount of bound FAD [9]. In that case, the exponential coefficients of both NAD(P)H and FAD were calculated from a biexponential fitting procedure. Whereas bound NAD(P)H is correlated to the amplitude of the longer lifetime component a_2_, a reverse trend is followed for FAD. The fluorescence lifetime of the protein-bound FAD is shorter as compared to the free FAD due to the efficient fluorescence quenching of the isoalloxazine chromophore by adenine, induced by the folding of FAD [20,21]. Therefore, the FLIRR index is defined as NAD(P)Ha_2_/FADa_1_, where FADa_1_ is the amplitude of the shorter lifetime component of FAD.

The bound NAD(P)H increases during OXPHOS; however, the situation for FAD is very complicated. There are reports suggesting that FAD bound to proteins is not changed or diminished during OXPHOS, which in addition to the increased protein-bound component of NAD(P)H might contribute to a higher FLIRR value [9]. In correlation, inhibiting complex II of the respiratory chain during Parkinson’s disease induced an increased concentration of FAD bound to proteins [22]. However, the reverse was also found. In a work by Skala et al., [23] more glycolysis was found in high grade compared to low grade and normal tumor, which was correlated with a decrease of bound NAD(P)H. Simultaneously, the relative amount of bound FAD also decreased, which could induce a higher FLIRR index, in contrast to the expected decrease during glycolysis. However, results can differ significantly and be compared only if the same experimental conditions are maintained (excitation wavelength, emission filters, etc.). Moreover, FAD contributes to various dehydrogenase systems [24]. Even for free FAD in aqueous buffer solution, a heterogeneous fluorescence intensity decay with two major lifetime components was reported where a dominant 7 ps component that is characteristic of ultrafast fluorescence quenching and a 2.7 ns component interpreted as moderate fluorescence quenching was found [14,25,26]. Moreover, the fluorescence decay has been attributed to two molecular conformations as stacked and opened forms [27]. Also, FAD fluorescence lifetime was found to be pH dependent [26,28], which increases the complexity of interpretation inside cells.

The consideration of FAD FLIM is complicated enough; however, one important component was neglected so far—namely, contributions from FMN during FAD detection. As demonstrated in Figure 1, a subgroup in the FAD molecular structure is FMN, the mononucleotide. Whereas FAD with respect to FLIRR mainly has its function in complex II of the respiratory chain, FMN is involved in complex I. Therefore, FMN that shows opposite fluorescence lifetime characteristics with an exceptionally long lifetime for bound FMN compared to FAD must be considered when investigating metabolic FLIM. Due to this, difficulty can occur in the interpretation of FLIRR. One has to be aware that although concentration of FMN is normally below FAD, the ratio depends on the cell type (see reference [29]) and the fluorescence quantum yield of FMN is approximately a factor of 10 times higher as compared to FAD [30]. Therefore, FMN was considered during advanced metabolic FLIM within this work. For this reason, we extended the FLIRR index approach and distinguished FLIRR1, FLIRR2 and FLIRR3, after a three-exponential fitting of the flavin signal. The definition of the ratios are as follows:

$$\mathrm{FLIRR}1=\frac{\mathrm{NAD}\left(P\right)H_{a_{2}\%}}{{\mathrm{FAD}}^{+}{\;}_{a_{1}\%}}$$

$$\mathrm{FLIRR}2=\frac{\mathrm{NAD}\left(P\right)H_{a_{2}\%}}{{\mathrm{FAD}}^{+}{\;}_{a_{2}\%}}$$

$$\mathrm{FLIRR}3=\frac{\mathrm{NAD}\left(P\right)H_{a_{2}\%}}{{\mathrm{FAD}}^{+}{\;}_{a_{3}\%}}$$

FLIRR1 relates bound NAD(P)H to bound FAD (interpreted as shortest lifetime component), FLIRR2 bound NAD(P)H to free FAD (interpreted as mean lifetime component) and FLIRR3 bound NAD(P)H to bound FMN (interpreted as longest lifetime component). In addition, we compared the FLIRR results with the metabolic index:

$$\frac{\mathrm{NAD}\left(P\right)H_{a_{1}\%}}{\mathrm{NAD}\left(P\right)H_{a_{2}\%}}$$

and determined the significance during metabolic imaging of keratinocytes and squamous carcinoma cells.

## 2. Results and Discussion

**Figure 2:**
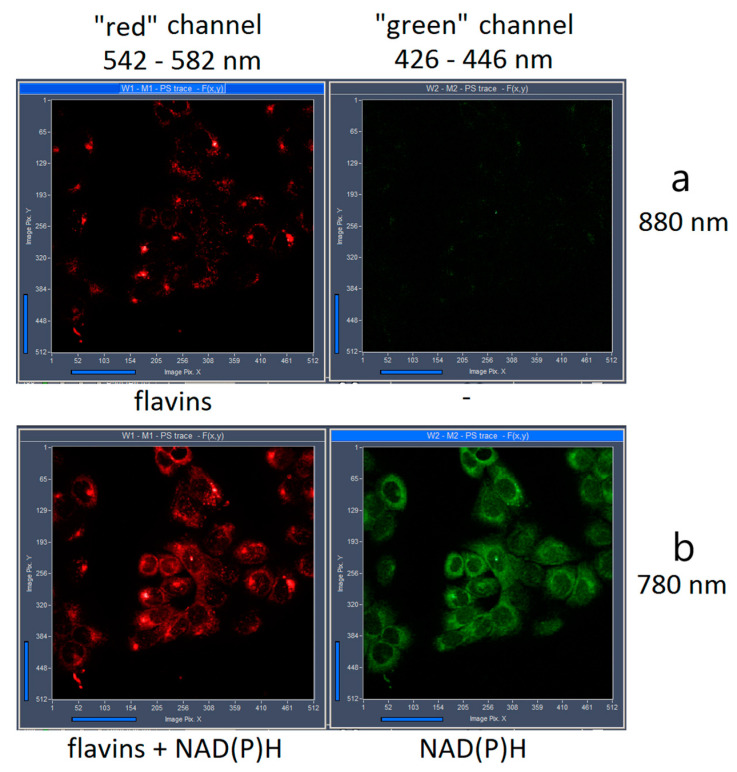
Intensity preview images obtained during lifetime measurements after two-photon excitation at 880 nm (**a**) and 780 nm (**b**) of HaCaT cells. Fluorescence was detected in the spectral range 426–446 nm (green channel) and 542–582 nm (red channel).

The emission of different intracellular flavin molecules were detected within the spectral range between 542–582 nm (see Figure 2, “red” channel) after two-photon excitation at 880 nm. The overlapped emission spectra of FMN and FAD including their protein-bound und free states does not allow for spectrally separating the coenzyme signal. The measured autofluorescence decay furthermore demonstrated complex multiple exponential kinetics. In fact, we differentiated at least three fluorescent components with different lifetimes for HaCaT, as well as SCC4 cells using a three-exponential incomplete model (see Table 1):

I(t) = ∑_i_ a_i_ exp(−t/τ_i_)

$$\left(I\right)=\sum_{i}a_{i}\exp\left(-t/\tau_{i}\right),$$

where τ_i_ and a_i_ denote the fluorescence lifetime of component i and its exponential coefficient, respectively. χ^2^ values were maintained at below 1.1. The three components within the observed spectral range were assigned to protein-bound FAD, free FAD, and FMN. The shortest lifetime and fast decaying component τ_1_ with the highest exponential coefficient a_1_ were correlated to the FAD cofactor that is bound to the mitochondrial enzymes. It is likely that a different protein environment of FAD within the cancerous SCC4 and normal HaCaT cells contribute to the significant difference of the τ_1_ values (*p*-value = 9.75 × 10^−5^), presented in Table 1.

To measure the lifetime of free FAD, we investigated the fluorescence decay of 1 mM buffer solution of FAD at 37 °C after two-photon excitation at 880 nm within the emission range 542–582 nm. The fluorescence intensity decay curve was fitted using a mono-exponential incomplete model, leading to a fluorescence lifetime of 1.90 ± 0.6 ns with χ^2^ = 1.3 that closely resembles the value of 1.7 ns reported in the literature [14]. The cited work, along with our results, supports the assumption that the lifetime component τ_2_, which we calculated within our cells (see Table 1), can be assigned to intracellular free FAD. In detail, the mean fluorescence lifetime τ_2_ in the cells was 1.53 ± 0.09 ns for HaCaT and 1.40 ± 0.10 ns for SCC4 cells with the corresponding coefficients a_2_ = 23.18 ± 1.69% and 21.94 ± 0.85% for an incomplete three-exponential free fitting procedure (see Table 1). Moreover, FMN could also contribute to τ_2_. Berg and coworkers [14] reported that the fluorescence lifetime of FMN consists of two components with 1.5 (1.3–1.7) ns and 4.7 (4.7–4.8) ns with the relative amplitudes 12% and 88%. The component at 1.5 ns was attributed to free FMN, whereas the component with the long lifetime around 4.7 ns correlates with protein bound FMN. The third component of our incomplete three exponential fit showed a mean lifetime of 5.40 ± 0.54 ns for HaCaT and 4.87 ± 0.46 ns for SCC4 cells (see Table 1). As mentioned, the long lifetime was also observed by others for intracellular FMN [14,31,32]. However, as described by Esposito et al. for FAD, a three exponential fit can also be obtained in solution that is pH dependent, which can be explained as a mixture of stacked-unstacked species [27]. The situation is therefore quite complex, and interpretations have to be made with care.

In Table 1, a significant difference of the mean flavin fluorescence lifetime for the two cell types is presented (*p* = 9.02 × 10^−7^). Also, the first component τ_1_, as well as the second component τ_2_ of the fitting model that we ascribed to protein-bound and free FAD was significantly different (*p*-value < 0.001). However, the amplitudes (a_i_) that correspond to the contributions of the different decaying components showed less significant difference between the two cell types. It is known that fluorescence lifetime fixation during fitting of the fluorescence decay results in valid calculations [1,33], so we made an attempt to apply an incomplete three-exponential decay model using the fixed lifetime values τ_1_ = 250 ps, τ_2_ = 1400 ps and τ_3_ = 5000 ps. These values were based on the calculations of Table 1, as well as the aforementioned published data. The results of the fitting model with fixed lifetimes are shown in Table 2. Now a_1_, a_2_ and a_3_ were statistically significantly different for HaCaT and SCC4 cells (*p*-value = 5.45 × 10^−5^, 5.56 × 10^−5^ and 4.12 × 10^−5^, respectively). This result exhibits the individual contributions of the three decaying components of flavins inside different cells.

In addition to FAD, we investigated FLIM of NAD(P)H in order to calculate the metabolic state of the cells. The “green” spectral channel (426–446 nm) was used to detect the fluorescence decay of protein-bound and free NAD(P)H. Two-photon laser excitation was performed at 780 nm for NAD(P)H. Also, the flavins are excited at the same wavelength, but emission of flavins was detected solely within the “red” (542–582 nm) spectral channel of our FLIM system, without any contribution in the “green” channel (see Figure 2). The mean lifetime of NAD(P)H in HaCaT and SCC4 cells was calculated using a two-exponential incomplete fitting with the fixed components τ_1_ = 400 ps and τ_2_ = 2.5 ns corresponding to free und protein-bound NAD(P)H, respectively [33,34]. As demonstrated in Table 3, τ_mean_ was significantly different for the two cells. In addition, the NAD(P)H metabolic index a_1_/a_2_ was significantly different (*p* = 6.07 × 10^−9^) and higher in case of the tumor cells. This correlates with increased glycolytic metabolism, described recently in the literature [16,17,18].

The FLIRR index NAD(P)Ha_2_/FADa_1_ [9], which relates bound NAD(P)H to bound FAD was calculated for HaCaT as well as SCC4 cells. In addition to the redox ratio index defined by Periasamy and coworkers, we introduced FLIRR1, FLIRR2 and FLIRR3, as FLIRR1 = NAD(P)Ha_2_/FADa_1_, FLIRR2 = NAD(P)Ha_2_/FADa_2_ and FLIRR3 = NAD(P)Ha_2_/FADa_3_ (see Section 3.2). Obviously FLIRR1 corresponds to the former FLIRR index. The values are presented in Table 4 for a three exponential fitting procedure of the flavin decay with free lifetimes and a two-exponential fit of NAD(P)H with fixed lifetimes. In addition, the flavin decay was also analyzed using the fixed lifetime values: τ_1_ = 250 ps, τ_2_ = 1.4 ns and τ_3_ = 5 ns. The results for the respective FLIRR indices are presented in Table 5. FLIRR1 was significantly different for both fitting procedures (free and fixed flavin lifetimes), whereas FLIRR2 and FLIRR3 revealed significance only for the free fit. In addition, the FLIRR1 index derived with free fitting of the flavin lifetimes (*p* = 3.89 × 10^−9^) was statistically more significant than the NAD(P)H metabolic index (*p* = 6.07 × 10^−9^), where only NAD(P)H is considered. This underlines the importance of investigating both NAD(P)H and FAD for the evaluation of metabolic differences between tumor and normal cells.

**Figure 3:**
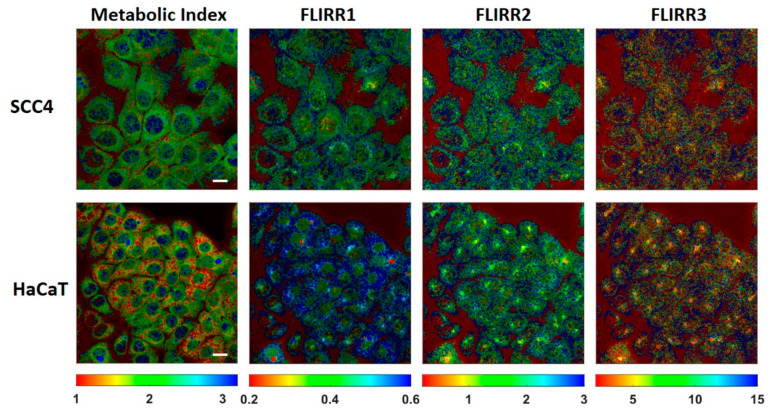
Images of the intracellular distribution of the metabolic NAD(P)H index and FLIRR1, FLIRR2, FLIRR3 indices within HaCaT and SCC4 cells. Scale bar 20 µm.

Figure 3 demonstrates the intracellular distribution of the metabolic NAD(P)H index and the different FLIRR indices following the fitting procedures described in Table 3 and Table 4. Thus, for NAD(P)H, a two-exponential fitting procedure with fixed lifetimes and for FAD a three-exponential free lifetime fit was used. For the FLIRR images, the pixel information of two different independent spectral channels must be calculated, which was done using a Matlab program (see Section 3.2). The images of the metabolic NAD(P)H index and FLIRR1 reveals clear different lifetime distribution histograms between the two cell lines, whereas no notable differences in case of FLIRR2 and FLIRR3 were found. Compared to the metabolic NAD(P)H index, the image of FLIRR1 demonstrates a clear difference for the two cell lines not only with respect to the mean numbers, but also to the distribution pattern within the cells.

In conclusion, we could clearly demonstrate significant differences of both metabolic NAD(P)H index and the FLIRR1 index in HaCaT as compared to SCC4 cells. In order to demonstrate if this is valid in a cell model with more complexity, we cocultured HaCaT and SCC4 cells as described in materials and methods.

**Figure 4:**
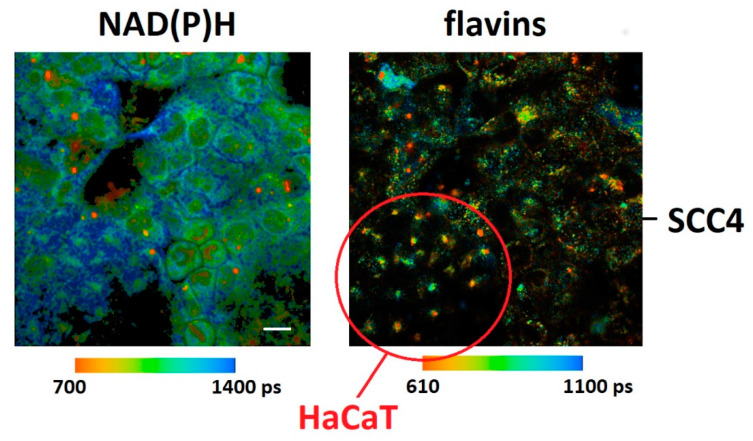
Fluorescence lifetime images of co-cultured HaCaT and SCC4 cells. The autofluorescence of NAD(P)H was recorded in the “green” (426–446 nm) spectral channel under 780 nm two-photon excitation whereas the autofluorescence of flavins was collected in the “red” (542–582 nm) spectral channel under 880 nm two-photon excitation. Scale bar 20 µm.

**Figure 5:**
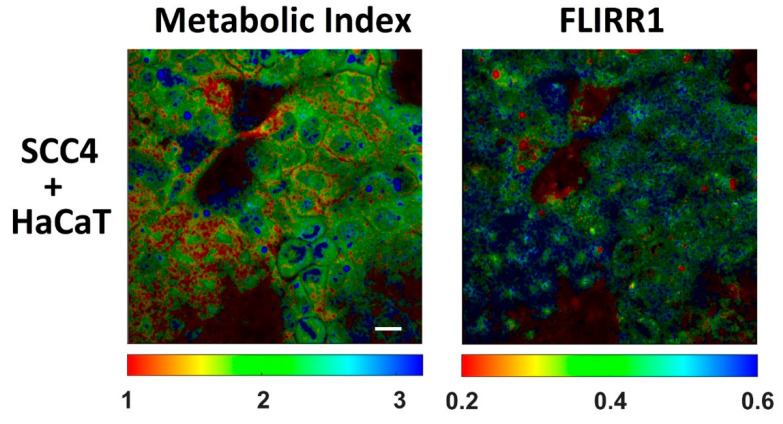
Intracellular distribution of the metabolic index and the redox ratio FLIRR1 within co-cultured HaCaT and SCC4 cells. Scale bar 20 µm.

Figure 4 demonstrates FLIM images of NAD(P)H and flavins in HaCaT/SCC4 cocultures. Cells could also be distinguished by their morphological appearances and lifetime distribution. The red circle indicates a cluster of HaCaT cells where a punctuated fluorescence was observed in the flavin channel. In correlation with the results in monocultures, the mean lifetime of NAD(P)H was significantly longer in HaCaT as compared to SCC4 cells. The difference of the mean lifetime of flavins was less significant (see Table 6). From Table 6, the metabolic index was significantly decreased for the HaCaT cells (*p* = 0.012). Also, the increase of the FLIRR1 index was significant (*p* = 0.007), which correlates with the results in monocultures. Figure 5 demonstrates images of the intracellular distribution of the metabolic NAD(P)H index and the FLIRR1 index. A Matlab routine was described in materials and methods which was used to calculate the FLIRR1 image. Both imaging procedures enabled a clear discrimination between the different cell types. A punctuated appearance was found within the FLIRR1 distribution in the HaCaT cells.

## 3. Materials and Methods/Experimental

### 3.1. FLIM of NAD(P)H and Flavins

The FLIM technique for NAD(P)H and flavins was based on multiphoton laser scanning microscopy in combination with advanced multidimensional TCSPC technique. A femtosecond pulsed Mai Tai AX HPDS titanium-sapphire laser (Spectra Physics, Darmstadt, Germany) was used for two-photon excitation. The laser has a repetition rate of 80 MHz and a tuning range from 690 to 1040 nm. The maximum optical output power was about 3 W at 800 nm, and the temporal pulse width was below 100 fs. The laser pulses were modulated by controlling the optoacoustic modulator (AOM) of the laser scanning microscope (LSM 710 NLO, Carl Zeiss, Jena, Germany) by a TCSPC system generated signal.

The non-descanned detection (NDD) port of the microscope was used for the signal detection. The fluorescence signals were separated by the beam-splitter LP 490. The band-pass filters 436/20 nm and 562/40 nm (AHF Analysentechnik, Tübingen, Germany) were used to filter the emission signals. In regard to the installed emission BP filter 355–690 nm, we have defined the spectral channels as 426–446 nm (“green” channel) and 542–582 nm (“red” channel) and used them for the detection of NAD(P)H and flavin fluorescence, respectively. The HPM-100-40 hybrid detectors (Becker & Hickl GmbH, Berlin, Germany) with a detection wavelength range from 300–700 nm were used in both channels.

The autofluorescence originating from NAD(P)H and flavins was excited by two-photon excitation at 780 nm and 880 nm, respectively. Since Mai Tai AX HPDS titanium-sapphire laser does not emit two excitation wavelengths simultaneously, we performed FLIM measurements consecutively and not concurrently with two times scanning of the investigated cell area. Firstly, the flavins were excited at 880 nm, which was followed by laser irradiation at 780 nm, which excites both flavins and NAD(P)H (see Figure 2). This order of excitation has been chosen because it induces minimal impact on the cells. Also, the reverse order of excitation (first 780 nm and then 880 nm) did not change the results significantly (data not shown).

The laser power of the microscope was reduced by the internal AOM of the LSM 710 and measured to be 10 mW in the sample plane. A resolution of 512 × 512 pixels with 256 time-channels in each pixel was selected for the primary data collection. The scanning was performed at a frame time of 7.75 s that corresponds to a pixel dwell time of 6.30 µs. The total acquisition time was maintained at 1 min. The objective lens used was an EC Plan-Apochromat 40×/1.3 oil (Carl Zeiss, Germany) with a scan area of 212.1 µm × 212.1 µm and zoom 1.

### 3.2. FLIM Data Analysis

The fluorescence lifetime imaging data were collected in SPCM64 software and thereafter sent to SPCImage software (version 8.1, Becker & Hickl GmbH, Berlin, Germany) for processing. The FLIM data of the NAD(P)H autofluorescence within the “green” (426–446 nm) spectral channel and the flavins autofluorescence within the “red” (542–582 nm) spectral channel (see Figure 2) were analyzed by fitting the fluorescence decay curve to a multi-component exponential incomplete model with a WLS fit method. For NAD(P)H, a two-exponential incomplete model I(t) = a_1_ e^−t/τ_1_^ + a_2_ e^−t/τ_2_^ was used, where I(t) is the fluorescence intensity at time t, τ_1_ and τ_2_ are the fluorescence lifetimes of the first and second component, corresponding to unbound and bound NAD(P)H, a_1_ and a_2_ are the associated relative amplitudes. For the estimation of the mean lifetime (τ_mean_) calculated as (a_1_ × τ_1_ + a_2_ × τ_2_)/(a_1_ + a_2_), the fixed lifetimes τ_1_ = 400 ps and τ_2_ = 2500 ps were chosen in accordance with literature reports [33]. For the flavin’s autofluorescence, a three-exponential incomplete model I(t) = a_1_ e^−t/τ_1_^ + a_2_ e^−t/τ_2_^ + a_3_ e^−t/τ_3_^ was used. τ_1_ was correlated to bound FAD, τ_2_ to unbound FAD and τ_3_ mainly to bound FMN; a_1_, a_2_ and a_3_ are the relative amplitudes. In all calculations, the total number of photons per trace was increased by maintaining a spatial binning factor of 2.

To establish the metabolic index and FLIRR distribution images, the relative amplitudes of the decay components and the photon counts per pixel of the respective FLIM data was exported from the SPCImage software after image analysis of the NAD(P)H and FAD channels as two-dimensional data matrices and imported in the Matlab software (The MathWorks Inc., Natick, MA, USA; Version 2018b) for the generation of the false color images. The method to generate the metabolic index and FLIRR false color images is described in the Supplementary Figure S1.

The metabolic index and extended FLIRR indexes were calculated by means of implementing the following formulas in Matlab:

$$\mathrm{Metabolic}\;\mathrm{Index}=\frac{\mathrm{NAD}\left(P\right)H_{a_{1}\%}}{\mathrm{NAD}\left(P\right)H_{a_{2}\%}}$$

$$\mathrm{FLIRR}1=\frac{\mathrm{NAD}\left(P\right)H_{a_{2}\%}}{{\mathrm{FAD}}^{+}{\;}_{a_{1}\%}}$$

$$\mathrm{FLIRR}2=\frac{\mathrm{NAD}\left(P\right)H_{a_{2}\%}}{{\mathrm{FAD}}^{+}{\;}_{a_{2}\%}}$$

$$\mathrm{FLIRR}3=\frac{\mathrm{NAD}\left(P\right)H_{a_{2}\%}}{{\mathrm{FAD}}^{+}{\;}_{a_{3}\%}}$$

A colormap was applied to the resulting matrices, each representing one index described above to create false color images. The false color images were then superimposed with the inverted intensity images (photon counts) from the 780 nm excitation NAD(P)H channel to generate a metabolic index distribution image and from 880 nm excitation flavins channel to generate all FLIRR distribution images.

### 3.3. Cell Culture Studies

We analyzed the FLIM data, FLIRR and the redox state within two different cell models, the human oral squamous carcinoma cell SCC4 (ATCC-Nr. CRL-1624) and the immortalized keratinocyte cell line HaCaT. SCC4 are reported to be fatty acid induced chemotherapy resistant and invasively growing [35], were cultivated in a nutrient mixture F-12 medium (DMEM/F-12, Thermo Fisher Scientific, Waltham, MA USA) supplemented with 1% GlutaMAX™ (Thermo Fisher Scientific, Waltham, MA USA), 400 mg/mL hydrocortisone (Sigma-Aldrich, St. Louis, MO, USA) and 10% fetal bovine serum (FBS) (Biochrom GmbH, Berlin, Germany) at 37 °C and 5.0% CO_2_. For this kind of tumor, a glycolytic mechanism is expected which we tried to confirm by the FLIRR analysis. On the other hand, we cultivated the spontaneously transformed aneuploid immortalized keratinocyte cell line obtained from the adult healthy human skin HaCaT, which was grown in DMEM with 4,5 g/L glucose (Thermo Fisher Scientific, Waltham, MA, USA) supplemented with 1% GlutaMAX™ and 10% fetal bovine serum (FBS) at 37 °C and 5.0% CO_2_. For microscopy, the cells were seeded on the glass bottom of a 24-well dish (Cellvis, Sunnyvale, CA, USA) at a cell density of 2 × 10^4^ cells/well for SCC4 and 1 × 10^4^ cells/well for HaCaT. The cells were grown for 48 h in the incubator at 37 °C and 5.0% CO_2_. Microscopic measurements were performed at 37 °C in Tyrode’s buffer (135 mM NaCl, 5 mM KCl, 1 mM MgCl_2_, 1.8 mM CaCl_2_, 20 mM HEPES, 5 mM glucose, pH 7.4).

In addition to the monocultures, we cocultured the HaCaT and SCC4 cells, seeding them sequentially in 24-well dishes on glass bottom at a density of 1 × 10^4^ cells each. Before seeding, the wells were coated for 60 min at 37 °C with poly-L-lysin. For the cocultures, DMEM medium with 4.5 g/L glucose supplemented with 1% GlutaMAX™ and 10% FBS was used. The cells were grown for 48 h in the incubator at 37 °C and 5.0% CO_2_. Microscopic measurements were performed at 37 °C in Tyrode’s buffer.

### 3.4. Statistical Analysis

The statistical evaluation was performed using the software Origin(Pro), 2019b (64-bit, 9.6.5.169) (OriginLab Corporation, Northampton, MA, USA). First, a Shapiro–Wilk normality test was used to check the Gaussian distribution of the datasets. For all FLIM data, a normal distribution was checked and confirmed. Thereafter, a one-way ANOVA test with Bonferroni mean comparison tests were used for the determination of statistical significance between the mean of datasets from two different live cell lines. The statistically significant normal distributions of two populations were predefined with a significance level of 0.05. For the data analysis, we used three different samples from three independent measurement days to obtain a total of 16 fluorescence lifetime imaging measurements for every spectral channel in each live cell type. Each fluorescence lifetime image contained approximately 40–60 live cells in case of HaCaT or 25–40 live cells in case of the larger SCC4. We estimated the fluorescence lifetime parameters such as mean lifetime (τ_m_), lifetime of each fluorescing component (τ_i_) and relative amount of each fluorescing component (a_i_) from the entire fluorescence lifetime image. The fluorescent signal includes fluorescence from all intracellular compartments such as cytoplasm, mitochondria, etc. (see Figure 3, Figure 4 and Figure 5). Since FLIM measurements of intrinsic autofluorescence are extremely susceptible to interference, we did not use additional counterstaining with further fluorescent dyes. Therefore, we applied FLIM measurements only to unstained cells.

## 4. Summary and Conclusions

Metabolic FLIM is now widely accepted to be one of the most powerful techniques to image bioenergetic status and changes in cells and tissue. However, the correct interpretation of the results could be difficult. Whereas an attribution of the lifetimes as an indicator for cell metabolism is mainly accepted, it is debated if this is valid for the redox state of cells. With respect to NAD(P)H FLIM this is true only for special conditions, if the NADH/NAD^+^ pool is stable and careful interpretation of the redox state is needed when comparing the fluorescence lifetime of different cell systems [3,4,5,6]. Therefore, new algorithms are developed to circumvent these problems and to image cell metabolism and redox state from fluorescence lifetimes in complex cellular systems.

Innovative ideas have been evaluated using the lifetime components of FAD together with NAD(P)H (see for example reference [2]). Recently, the fluorescence lifetime induced redox ratio (FLIRR) where FLIM results of NAD(P)H and FAD are correlated were successfully introduced [9]. It was demonstrated that in the FLIRR index, the relative amount of bound NAD(P)H divided by the relative amount of bound FAD increases during OXPHOS and correlates with a higher oxidative state. However, contradictory results were also reported, claiming detailed interpretations. FLIM of FAD could be especially complicated. In contrast to NAD(P)H, the fluorescence lifetime of the protein-bound FAD is shorter compared to the free FAD [20,21]. For free FAD in aqueous buffer solution, a heterogeneous fluorescence intensity decay with two major lifetime components was reported—a dominant ultrafast 7 ps component and a 2.7 ns component interpreted as moderate fluorescence quenching [14,25,26]. Moreover, the fluorescence decay has been attributed to two molecular conformations as stacked and opened forms and FAD fluorescence lifetime was found to be pH dependent that increases complexity of interpretation inside cells [26,28]. Depending on the fitting procedure and experimental conditions, different lifetime components and values were found for FAD in various cell types. Whereas in prostate cancer cells two-exponential fitting led to two components with 120 ps and 3.38 ns [9], the lifetime τ_1_ for protein-bound FAD and τ_2_ for free FAD in Kasumi-1 cells was reported to be around 400 ps and 2.4 ns, respectively [23]. In isolated cardiomyocytes, a three-exponential fitting model let to 200 ps, 870 ps and 2.81 ns, respectively, with associated amplitudes at 71.8, 17.2, and 11.0 [11]. In general, the lifetime of protein-bound FAD was reported to be within 80–700 ps for living cells [9,11,26]. Within our measurements, high values of the pre-exponential factor a_1_ which shows the relative contribution of the first component of the fitting model, supports the correlation with bound FAD, as FAD mostly exists as a part of flavoproteins in living systems [36,37].

From the discussion above, FAD FLIM is found to be a complex and correct interpretation of metabolic parameters can be challenging. Moreover, one component was neglected so far; namely, contributions from FMN during FAD detection. Our aim was therefore to investigate the simultaneous evaluation of the fluorescence lifetime of NAD(P)H, FAD and FMN for advanced metabolic FLIM. Where FAD with respect to FLIRR mainly has its function in complex II of the respiratory chain, FMN is involved in complex I. FMN shows reverse fluorescence lifetime characteristics as compared to FAD, with an exceptionally long lifetime for protein bound FMN (see Figure 1) which must be considered in the metabolic FLIM investigation. Although the concentration of FMN is normally below FAD, the ratio depends on the cell type [29] and the fluorescence quantum yield of FMN is approx. 10 times higher as compared to FAD [30]. Interestingly, the estimation of the fluorescence brightness seen in the FAD channel is not dominated by protein bound FAD. In contrast, the fluorescence intensities (q-values) were highest for FMN, with a significant difference between the two cells (data not shown). Due to this, we extended the FLIRR index approach and evaluated FLIRR1, FLIRR2 and FLIRR3 after three-exponential fitting of the flavin signal. FLIRR1 relates bound NAD(P)H to bound FAD, FLIRR2 bound NAD(P)H to free FAD and FLIRR3 bound NAD(P)H to FMN. The significance of the result was compared to the metabolic NAD(P)H index that relates free NAD(P)H to bound NAD(P)H. We could clearly demonstrate a significant difference between HaCaT keratinocytes and SCC4 cells for the metabolic NAD(P)H and FLIRR1 index in monocultures, as well as cocultures. FLIRR1 was significantly different for both fitting procedures (free and fixed flavin lifetimes), whereas FLIRR2 and FLIRR3 revealed significance only for the free fitting calculations. Moreover, the FLIRR1 index was statistically higher than the NAD(P)H metabolic index in monocultures, as well as cocultures. We conclude that FLIRR1 is the most significant in cocultures, although the NAD(P)H fluorescence lifetime is significantly different, but not the flavin lifetime. This underlines the importance of relating the exponential coefficients of both NAD(P)H and FAD for the evaluation of metabolic differences between tumor and normal cells. In addition, the images of FLIRR1 reveals a clear difference between the two cell lines, as evident from the calculated values (histogram), as well as the intracellular distribution pattern.

In summary, FLIRR1 was evaluated to demonstrate the most significant difference in metabolic imaging between HaCaT keratinocytes and SCC4 cells. This indicates that protein bound NAD(P)H and protein bound FAD mainly impacts the metabolic differences in both cell types. Although free FAD and FMN are also significantly different in the two cells (see Table 1 and Table 2), their relative contribution to the metabolic signal is small. However, this does not indicate that the fluorescence brightness seen in the FAD channel is dominated by the protein bound FAD. In contrast, as outlined above, the largest contribution originates from FMN that exhibits the highest fluorescence quantum yield.

## Figures and Tables

**Table 1:** Fluorescence lifetime values (τ_i_) and their corresponding exponential coefficients (a_i_) of flavins in HaCaT and SCC4 monocultures. The values were calculated by a three-exponential fit decay analysis.

	**τ_mean_ (ps)**		**τ_1_ (ps)**		**τ_2_ (ns)**		**τ_3_ (ns)**
HaCaT	791 ± 44		297 ± 33		1.53 ± 0.09		5.40 ± 0.54
SCC4	686 ± 52		246 ± 32		1.40 ± 0.10		4.87 ± 0.46
		**a_1_ (%)**		**a_2_ (%)**		**a_3_ (%)**	
HaCaT		71.62 ± 0.78		23.18 ± 1.69		10.34 ± 0.39	
SCC4		72.30 ± 0.88		21.94 ± 0.85		9.98 ± 0.79	

The statistical significance of the respective components between HaCaT and SCC4 cells was evaluated using one-way ANOVA test. The *p*-values were 9.02 × 10^−7^ for τ_mean_, 9.75 × 10^−5^ for τ_1_, 6.05 × 10^−4^ for τ_2_ and 6.11 × 10^−3^ for τ_3_ whereas for a_1_, a_2_ and a_3_ the p-value was 0.026, 0.014 and 0.112 respectively.

**Table 2:** Mean fluorescence lifetime (τ_mean_) and the associated exponential coefficients (a_i_) of intrinsic flavin autofluorescence in HaCaT and SCC4 monocultures, derived by an incomplete three-exponential fit decay analysis with fixed lifetime values: τ_1_ = 250 ps, τ_2_ = 1.4 ns and τ_3_ = 5 ns.

	τ_mean_ (ps)	a_1_ (%)	a_2_ (%)	a_3_ (%)
HaCaT	766 ± 47	68.7 ± 2.9	27.0 ± 2.6	4.98 ± 0.40
SCC4	706 ± 26	72.6 ± 1.6	23.5 ± 1.5	4.35 ± 0.34

The statistical significance of the respective components between HaCaT and SCC4 cell lines was evaluated using a one-way ANOVA test. The *p*-value was 1.05 × 10^−4^ for τ_mean_ and 5.45 × 10^−5^, 5.56 × 10^−5^, and 4.12 × 10^−5^ for a_1_, a_2_ and a_3_, respectively.

**Table 3:** Mean fluorescence lifetime (τ_mean_) and associated exponential coefficients (a_i_) of NAD(P)H in HaCaT and SCC4 cells. The values were calculated by a two-exponential fit decay analysis performed with the fixed lifetimes τ_1_ = 400 ps and τ_2_ = 2.5 ns.

	τ_mean_ (ps)	a_1_ (%)	a_2_ (%)	a_1/_a_2_
HaCaT	1160 ± 34	63.8 ± 1.6	36.2 ± 1.6	1.77 ± 0.12
SCC4	1073 ± 28	68.0 ± 1.3	32.0 ± 1.3	2.12 ± 0.13

The statistical significance of the respective components between HaCaT and SCC4 cell lines was evaluated using a one-way ANOVA test, that showed a *p*-value of 7.93 × 10^−9^ for τ_mean_, 6.07 × 10^−9^ for a_1_/a_2_, 8.14 × 10^−9^ for a_1_ and 7.58 × 10^−9^ for a_2_.

**Table 4:** FLIRR indices for HaCaT and SCC4 cells. FLIRR1 is the ratio between a_2_(NAD(P)H) and a_1_(flavins), FLIRR2 is the ratio of a_2_(NAD(P)H) and a_2_(flavins) and FLIRR3 is the ratio of a_2_(NAD(P)H) and a_3_(flavins). For the calculations, a three-exponential free lifetime fit decay analysis was performed for flavins and a two-exponential fixed lifetime fit decay for NAD(P)H with τ_1_ = 400 ps and τ_2_ = 2.5 ns.

	FLIRR1 (a_2/_a_1_)	FLIRR2 (a_2/_a_2_)	FLIRR3 (a_2/_a_3_)
HaCaT	0.51 ± 0.02	1.57 ± 0.14	3.5 ± 0.2
SCC4	0.44 ± 0.02	1.46 ± 0.08	3.2 ± 0.3

The statistical significance of the respective exponential components between HaCaT and SCC4 cell lines was evaluated using a one-way ANOVA test that showed a *p*-value of 3.89 × 10^−9^ for FLIRR1, 0.015 for FLIRR2 and 0.008 for FLIRR3.

**Table 5:** FLIRR indices of HaCaT and SCC4 cells derived by a three-exponential fit of the flavins with fixed lifetime values, τ_1_ = 250 ps, τ_2_ = 1.4 ns and τ_3_ = 5 ns. The NAD(P)H decay was fitted biexponentially using the fixed lifetimes τ_1_ = 400 ps and τ_2_ = 2.5 ns.

	FLIRR1 (a_2/_a_1_)	FLIRR2 (a_2/_a_2_)	FLIRR3 (a_2/_a_3_)
HaCaT	0.53 ± 0.04	1.35 ± 0.13	7.3 ± 0.7
SCC4	0.44 ± 0.02	1.37 ± 0.10	7.0 ± 0.7

The statistical significance of the respective components between HaCaT and SCC4 cell lines was evaluated using a one-way ANOVA test that showed a p-value of 4.70 × 10^−9^ for FLIRR1, 0.685 for FLIRR2 and 0.737 for FLIRR3.

**Table 6:** Mean fluorescence lifetime, metabolic index and FLIRR1 for NAD(P)H and flavins within co-cultivated HaCaT and SCC4 cells. NAD(P)H fluorescence decay was fitted biexponentially with fixed lifetimes τ_1_ = 400 ps and τ_2_ = 2.5 ns, the flavin fluorescence decay was fitted three exponentially with free lifetimes τ_i_.

	NAD(P)H τ_mean_ (ps)	Flavins τ_mean_ (ps)	Metabolic Index (NAD(P)H a_1_/NAD(P)H a_2_)	FLIRR1 (NAD(P)H a_2_/flavins a_1_)
HaCaT	1154 ± 51	704 ± 90	1.8 ± 0.2	0.49 ± 0.03
SCC4	1099 ± 47	673 ± 89	2.0 ± 0.2	0.46 ± 0.03

The statistical significance between HaCaT and SCC4 cell lines was evaluated using one-way ANOVA test. The *p*-value was 0.012 for the metabolic index and 0.007 for FLIRR1.

## Data Availability

The data presented in this study are available on request from the corresponding author. The data are not publicly available due to privacy.

## Supplementary Materials

The following are available online at https://www.mdpi.com/article/10.3390/ijms22115952/s1. Supplementary Figure S1. Flow diagram of workflow to establish the metabolic index and FLIRR 1, FLIRR 3 and FLIRR 3 false color images.

## Abbreviations

AOM	optoacoustic modulator
FAD	flavin adenine dinucleotide
FLIM	fluorescence lifetime imaging
FLIRR	fluorescence lifetime induced redox ratio
FMN	flavin mononucleotide
NAD(P)H	**reduced** nicotinamide adenine dinucleotide (phosphate)
OXPHOS	oxidative phosphorylation
TCSPC	time-correlated single photon counting
