# Supplementary material for: Bioenergetic Alterations of Metabolic Redox Coenzymes as NADH, FAD and FMN by Means of Fluorescence Lifetime Imaging Techniques
Source: Int J Mol Sci. 2021 May 31;22(11):5952. doi: 10.3390/ijms22115952 (PMC8199032; doi:10.3390/ijms22115952)
Supplement: Supplementary file 1 [file ijms-22-05952-s001.zip › ijms-1245637-supplementary.pdf]

International Journal of
Molecular Sciences

MDPI

Supplementary Materials

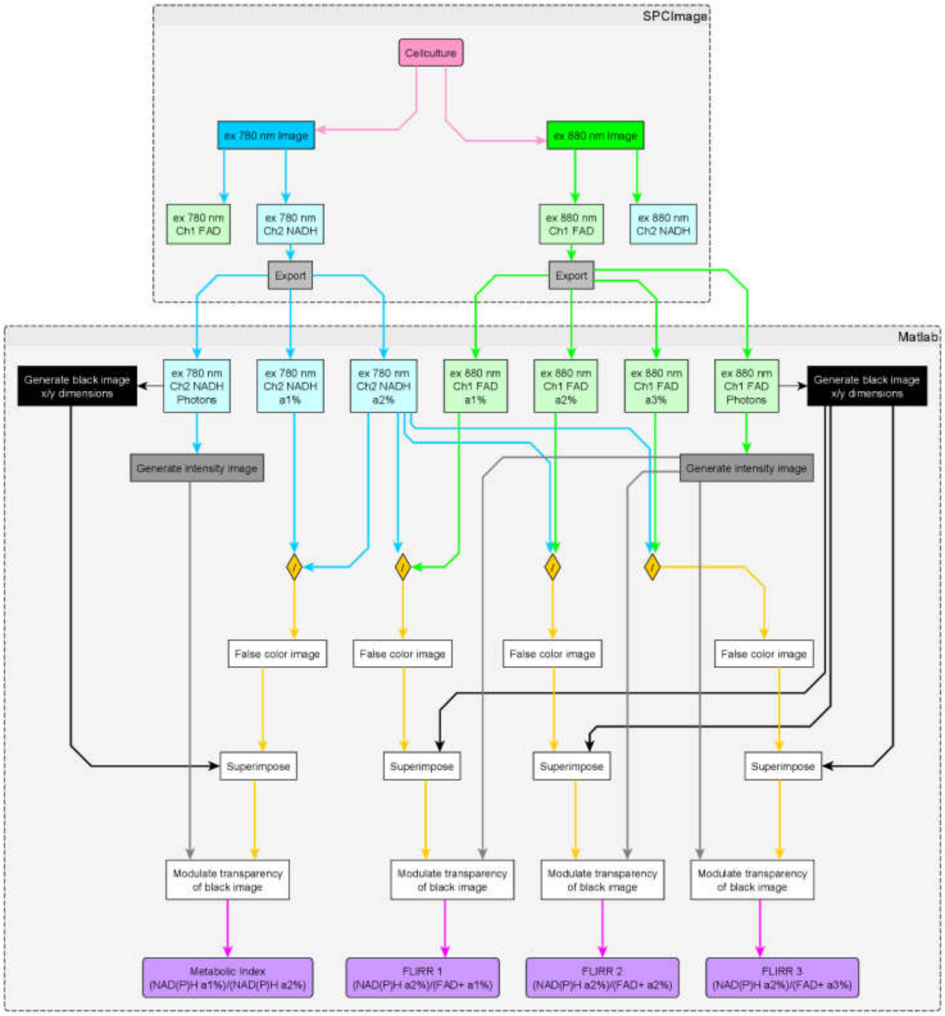

Supplementary Figure S1. Flow diagram of workflow to establish the metabolic index and FLIRR 1, FLIRR 3 and FLIRR 3 false color images. After completion of image analysis in SPCImage as described in section 3.2. "FLIM data analysis" the relative amplitudes of the fitfuncions to the NAD(P)H and FAD channels and the intensity image which represents the photon counts per pixel are exportet using the SPCImage export dialog. The exported data matrixes are importet into Matlab and the Metabolic Index and FLIRR 1 to 3 indizes calculated, according to the formulas described in section 3.2. A color map is applied to the resulting matrixes to create false color images which are then superimposed by the inverted intensity image to create the final images.
